# Assessment of Intracranial Collateral Circulation Using Novel TCCS Grading System in Patients With Symptomatic Carotid Occlusion

**DOI:** 10.3389/fneur.2020.00666

**Published:** 2020-07-24

**Authors:** Foad Abd-Allah, Haytham Rizk, Mohammad Ahmed Farrag, Mohamed Hafez Shaaban, Ahmed Nasreldein

**Affiliations:** ^1^Neurology Department, Kasr Alainy School of Medicine, Cairo University, Cairo, Egypt; ^2^Anatomy Department, Faculty of Medicine, Cairo University, Cairo, Egypt; ^3^Neurology Department, Assiut University Hospitals, Assiut University, Assiut, Egypt

**Keywords:** TCCS, cerebral, collaterals, total, carotid, occlusion, SPECT

## Abstract

**Objectives:** To establish a novel transcranial color-coded sonography (TCCS) grading system for collateral circulation in cases of symptomatic chronic total carotid occlusion (TCO), and to correlate this new grading system with cerebrovascular reserve capacity (CVR) measured by SPECT.

**Methods:** Thirty-four patients with symptomatic chronic TCO recruited from the neurovascular ultrasound laboratory of the department of Neurology at Cairo University Hospital during 3 years' time period and diagnosed by color-coded duplex were subjected to: clinical assessment, grading of cerebral collaterals using a proposed TCCS criteria, Brain SPECT studies at rest and with dipyridamole stress.

**Results:** The new grading system for cerebral collateral circulation showed a significant positive correlation with CVR (*P* < 0.001 and Spearman correlation coefficient 0.686).

**Conclusion:** The current study showed that this new TCCS grading system for cerebral collaterals is a reliable indicator for cerebral perfusion and reserve capacity in cases of chronic symptomatic TCO.

## Introduction

The clinical manifestations of occlusive carotid artery disease are highly variable with some patients diagnosed accidentally and others presenting with fatal ischaemic stroke. Twenty percent of ischaemic stroke patients have underlying significant carotid artery stenosis (1). The recurrent stroke risk in patients with total carotid artery occlusion (TCO) is ~6% per year, and this risk increases to up to 10% in patients with impaired cerebral blood flow reserve capacity even with the best medical treatment (2). In these patients, ischaemic stroke is due to either arterio-arterial embolus or hypoperfusion with failure of the cerebral collateral circulation to maintain adequate flow (3). The cerebral collateral circulation divides into primary and secondary collaterals, with the primary collaterals including the anterior communicating artery (ACOM) and posterior communicating artery (PCOM). The secondary collaterals include ophthalmic arteries and leptomeningeal vessels (4). This collateral circulation maintains sufficient perfusion to brain tissues distal to the site of arterial occlusion, and it therefore has important therapeutic and prognostic value (5). The use of imaging tools to assess these collateral circulations in patients with TCO has been the focus of extensive research for decades. Conventional cerebral angiography remains the standard method to evaluate collateral circulation; however, non-invasive methods of evaluating these collaterals, such as transcranial color-coded sonography (TCCS) and single-photon emission computed tomography (SPECT), are rapidly evolving. SPECT also allows the evaluation of regional cerebral perfusion and haemodynamic reserves (6, 7). This is especially important when evaluating patients with multi-vessel disease in whom the riskiest territory for urgent intervention must be identified. Patients with poor perfusion and reduced vasoreactivity are at risk for recurrent stroke (8).

## Materials and Methods

Cases were recruited from the neurovascular ultrasound laboratory of the department of Neurology at Cairo University Hospital during 3 years time period, January 1, 2014, to January 1, 2017. We prospectively studied patients with chronic TCO in either the common carotid artery (CCA) or the internal carotid artery (ICA) presenting by stroke or transient ischemic attack. Patients or their family members gave signed and informed consent after explanation of the study methods was provided. All patients were subjected to proper history taking (including bronchial asthma as a contraindication to dipyridamole injection), through neurological assessment, a full laboratory screening including renal functions to avoid contrast hazards, a complete cervical and intracranial ultrasound assessment with a high-resolution color-coded duplex sonography scanner (Philips HDI 5000) using a high-frequency (5- to 10-MHz) linear probe for the cervical arteries and a low-frequency (2- to 5-MHz) phased-array probe for the intracranial arteries. The examination was performed by experienced neurovascular sonographer certified by the Intersociety Commission for Certification in Neurosonology, the Neurosonology Research Group of the World Federation of Neurology (NSRG), and European Society of Neurosonology and Cerebral Hemodynamic (ESNCH). The CCA and ICA were examined using an examination protocol and interpreted according to the criteria published by the Society of Radiologists in Ultrasound 2003. ICA occlusion was diagnosed when occluding material was visualized in B-mode, color and pulsed Doppler signal was absent, and diastolic velocity in the ipsilateral CCA was low or absent. An intracranial vessel ultrasound examination was performed through the trans-temporal and the trans-orbital acoustic windows. The examined collaterals were the anterior communicating artery, posterior communicating artery, and ophthalmic artery. The neurovascular ultrasound findings were confirmed with at least one other modality like CT angiography, cerebral MRA, and a few cases got catheter-based angiography, but we didn't perform comparative study for every case with cerebral angiography.

We proposed the following collateral grading (CG) system based on existing collaterals (Table 1) where the collateral flow through anterior communicating artery (ACOM) was given 3 points while collateral flow through posterior communicating (PCOM) or ophthalmic arteries was given 1 point.

**Table 1:** Proposed collateral grading system.

**Description**	**Grade**
Collateral flow through all 3 vessels ACOM, PCOM, and OA	5
ACOM + 1 collateral flow through the PCOM or OA	4
Collateral flow through the ACOM only	3
Collateral flow through PCOM and OA	2
Collateral flow through the PCOM or OA	1
No collaterals	0

*ACOM, anterior communicating artery; PCOM, posterior communicating artery, OA: ophthalmic artery*.

### Single-Photon Emission Tomography (SPECT)

SPECT scan and dipyridamole scans were obtained at baseline and 48 h later under resting conditions according to the protocol of Te-Long et al. (9). The reconstructed SPECT images were assessed by a nuclear medicine consultant blinded to the patients' data. Images were evaluated for focal or regional decreases in tracer uptake, hemispheric uptake symmetry, and response to dipyridamole. Abnormal or asymmetric distribution included any area with <30% level of uptake or a clearly different or diminished uptake in a hemisphere or region. Any study that showed worsening of focal or hemispheric asymmetry or hypoperfusion after dipyridamole administration was interpreted as compromised perfusion reserve. Reduced regional cerebral blood flow (reduced rCBF) was defined as abnormal cerebral perfusion on a basal SPECT image. Reduced regional cerebrovascular reserve (reduced rCVR) was defined as abnormal cerebral perfusion that fell into a color range even lower than that obtained on basal SPECT after dipyridamole administration.

### Statistical Analysis

Pre-coded data were entered using “Microsoft Office Excel Software” (2013). Data were then transferred to the Statistical Package of Social Science Software (SPSS), version 21 for statistical analysis. Data were summarized using the mean, and the standard deviation for quantitative variables and frequency and percentage for qualitative variables.

Comparisons between groups were performed using independent sample *t*-test for quantitative variables and Chi square or Fisher's exact test for qualitative variables. Pearson or Spearman correlation coefficients were calculated to signify the association between quantitative or ordinal variables, respectively. Backward stepwise logistic and linear regression models (multivariate analysis) were conducted to identify the significant predictors of dependent qualitative and quantitative variables, respectively. *P* < 0.05 were considered statistically significant, and < 0.01 was considered highly significant.

## Results

We studied 34 patients including 26 males (76.5%) and 8 females (23.5%) with symptomatic chronic extracranial or intracranial TCO. Their mean age was 48.9 ± 10.4 (range, 24–65) years old. The baseline characteristics of the study group is explained in (Table 2), and none of our patients had a contralateral high grade carotid stenosis (≥50%).

**Table 2:** Baseline characteristics of the study population.

	**Description (*****n*** **=** **34)**	
	**N**	**%**
**Age**
<45	10	30
>45	24	70
Family history^*^
+VE	12	35.3
–VE	22	64.7
HTN^*^
+VE	23	67.6
–VE	11	32.4
DM^*^
+VE	15	44.1
–VE	19	55.9
**Dyslipidemia**
+VE	22	64.7
–VE	12	35.3
**Smoking**
+VE	21	61.8
–VE	13	38.2
**Etiology**
Atherosclerotic	27	79.5
Non-atherosclerotic	7	20.5
**Collateral grading**
Good (≥ 3)	26	76.5 %
Poor (<3)	8	23.5 %
CVR^*^
Impaired	12	35.3
Preserved	22	63.7

* *Family history for cerebrovascular disease HTN, Hypertension; DM, Diabetes Mellitus; CVR, cerebrovascular reserve*.

**Figure 1:**
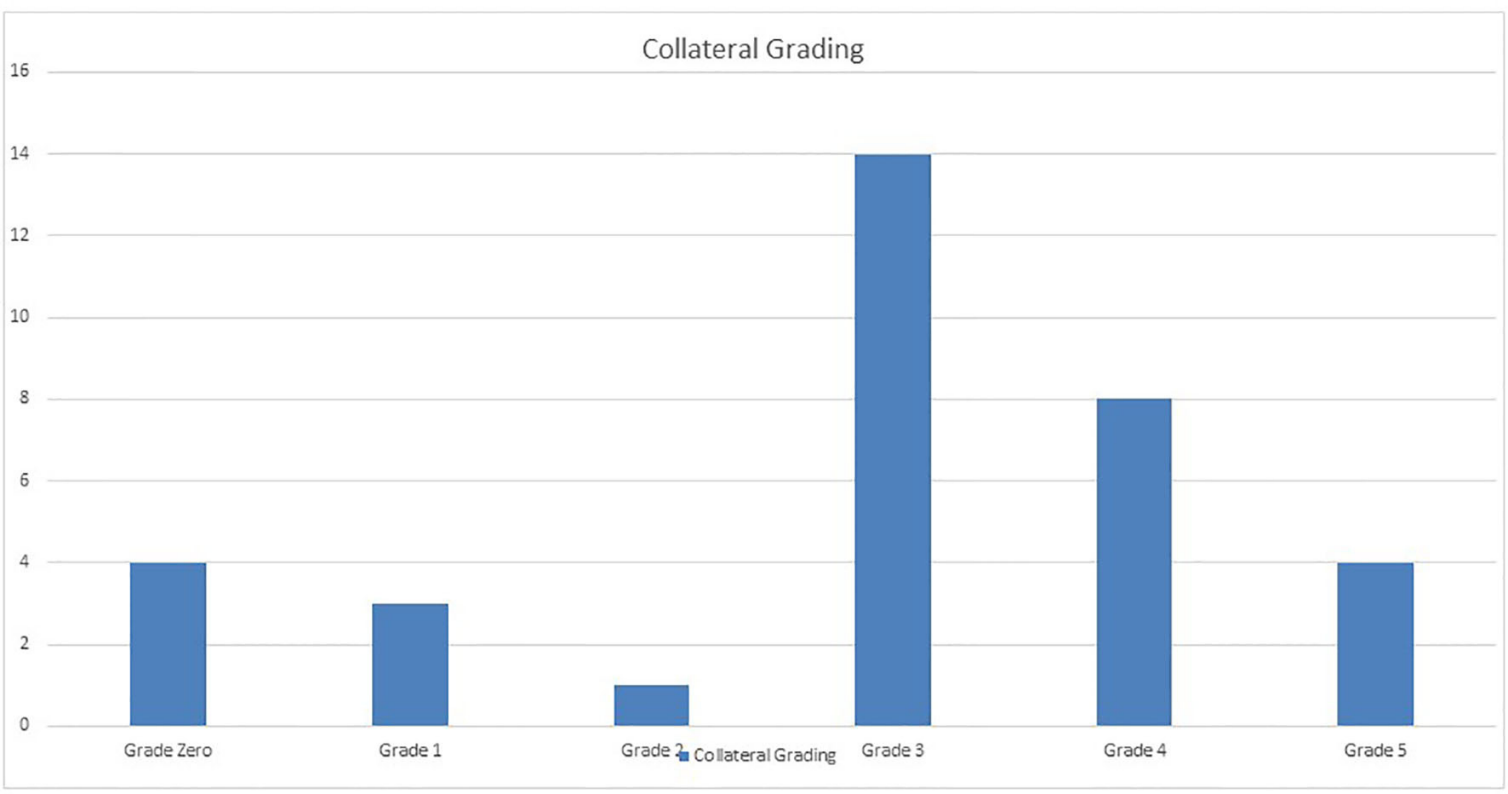
Distribution of collateral grading among the study population.

**Figure 2:**
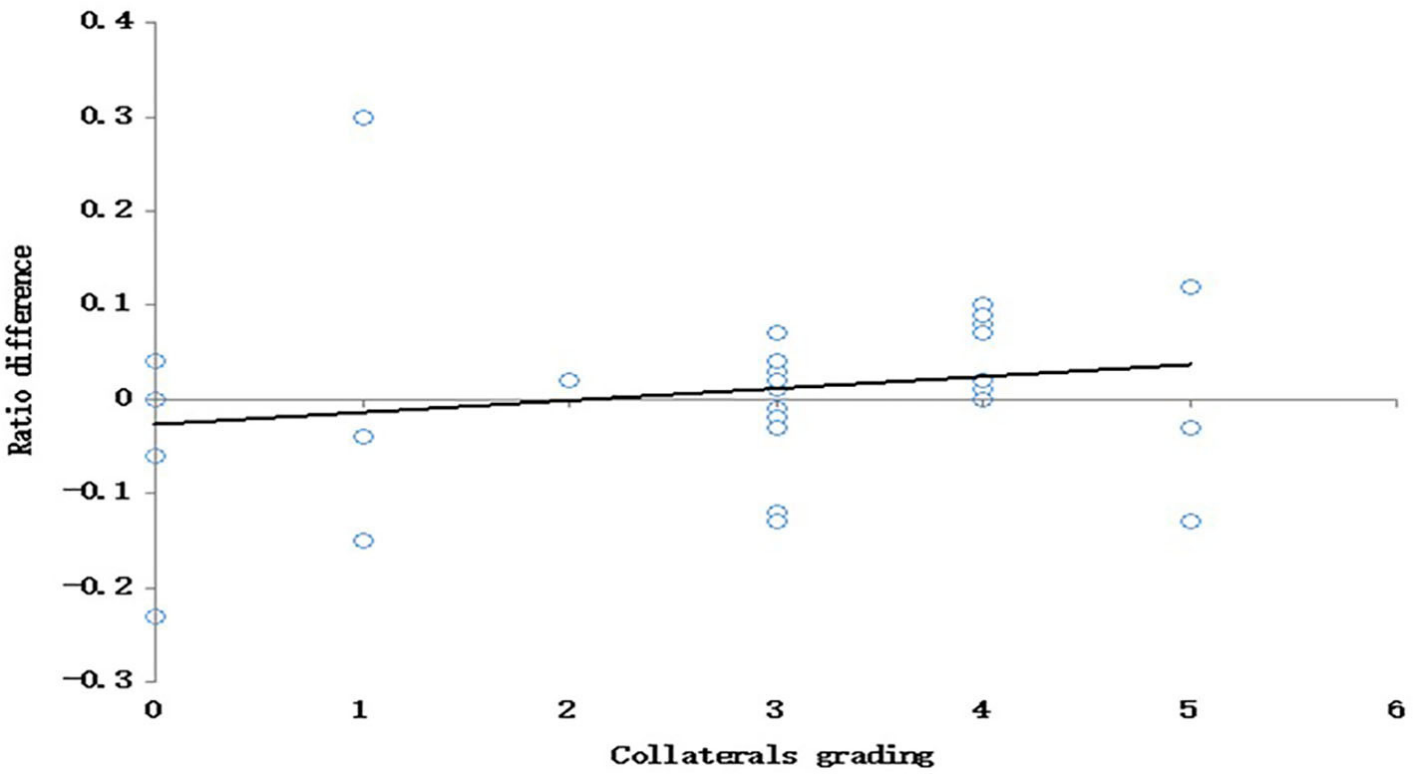
Correlation between CVR and the collaterals grading.

Collateral data were interpreted according to the proposed collateral grading system. The majority of cases had CG 3 (14 cases, 41%) (Figure 1). Twelve patients (35.3%) in the study group had impaired CVR, while 22 (63.7%) had preserved CVR. There was highly significant positive correlation between CVR and our novel collaterals grades with *P* < 0.001 and Spearman's correlation coefficient (0.686) (Figure 2).

Atherosclerosis was the most prevalent pathology among our study group and was found in 27 of the patients (79.4%); however, non-atherosclerotic pathologies were detected in 7 (20.6%) patients: dissection in 3 patients, Takayasu arteritis in 2 patients, Antiphospholipid syndrome in 1 patient, and radiation arteritis in 1 patient.

Patients with atherosclerotic carotid occlusion had a higher mean age than was found in non-atherosclerotic carotid occlusion patients (51.8 vs. 37.9 years old). Vascular risk factors were more prevalent in the atherosclerotic group than the non-atherosclerotic group. Good collaterals grades (≥3) were significantly more likely to be detected in the atherosclerotic group than in the non-atherosclerotic group (81 vs. 57%). Additionally more preserved CVR was found in the atherosclerotic group than in the non-atherosclerotic group (70 vs. 43%). Interestingly all 3 patients in the non-atherosclerotic group who had poor collateral grades and poor CVR also had carotid dissection.

## Discussion

**Figure 3:**
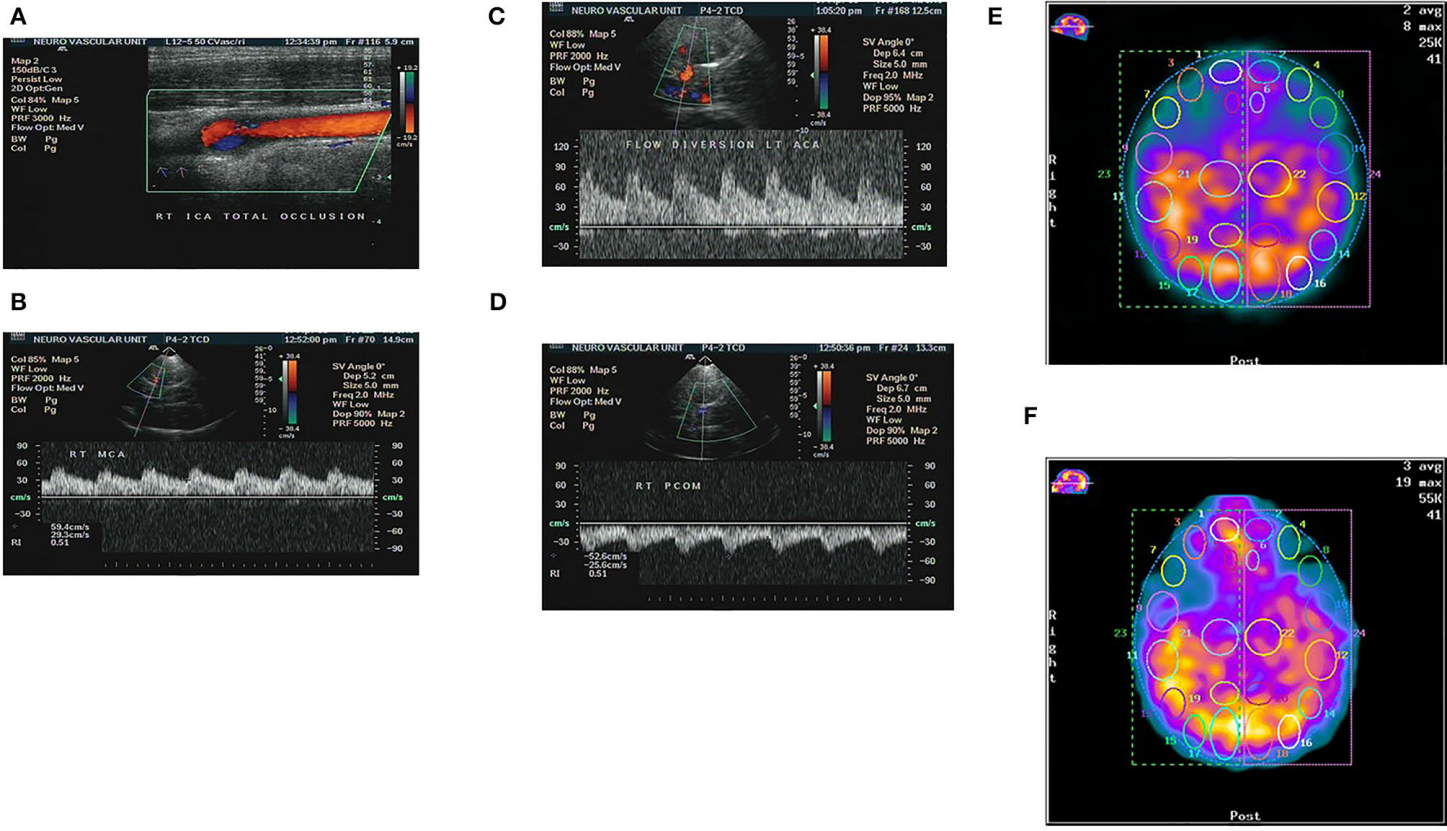
Patient with chronic total occlusion (CTO) of right ICA proved by color coded Doppler ultrasound **(A)**, and preserved flow in the ipsilateral right MCA due to compensatory flow from both anterior and posterior communicating arteries as detected by TCCS **(B–D)**. The same patient showed no sizable perfusion deficit at baseline SPECT study, Right hemisphere/left = 1.01 **(E)**, and no appreciable change after stress test, Right hemisphere/left = 0.98, denoting well-preserved CVR **(F)**.

The Cerebral collaterals play an important role in maintaining cerebral perfusion in patients with TCO. In our study, we propose a collateral grading system based on TCCD assessment, and then we analyzed the relationship between the proposed collateral grading system and CVR assessed by SPECT. The results of our study showed that there was positive relationship between our proposed grading system and CVR capacity measured by SPECT (Figure 3).

Our results are in agreement with those presented in previous reports such as Zarrinkoob et al. (10) who studied hemodynamic disturbance and compensatory pattern of collateral flow in patients with symptomatic carotid stenosis by studying blood flow rates (BFR) with four-dimensional phase-contrast magnetic resonance imaging and found that the ACOM was the major collateral that contributed to BFR equalization in patients with carotid artery stenosis.

Hartkamp et al. (11) studied the effect of carotid artery disease upon cerebral perfusion and cerebrovascular reactivity and found that haemodynamic impairment in the affected hemisphere was more severe in patients with posterior to anterior collateral flow through the PCOM than in patients with anterior collateral flow through the ACOM toward the middle cerebral artery MCA territory, which support our grading system.

Vernieri et al. (12) studied the relationship between the numbers and type of collaterals and both vasomotor reactivity (VMR), and stroke risk. They found VMR was normal and prognosis favorable in patients with full collateral development; among whom no patients experienced an ischemic stroke. However, the other group without collateral pathways showed impaired VMR, and an annual risk of ipsilateral stroke of 32.7%. Patients with 1 or 2 collateral pathways had a VMR values ranging from normal to strongly reduced. The ipsilateral stroke event risk was 17.5% in patients with 1 collateral vessel and 2.7% in patients with 2 collateral pathways. Data from this study highlight the significant role of collateral pathways in patients with steno-occlusive disease. Yamauchi et al. (13) studied the relationship between the pattern of collateral circulation and an increased oxygen extraction fraction (OEF), an indicator of inadequate collateral blood flow distal to the occlusion, and found that the presence of ophthalmic or leptomeningeal collaterals was associated with haemodynamic impairment in the form of increased OEF. Their results are consistent with our grading system. Sundaram et al. (14) found that patients with 2 or more collaterals (ipsilateral OA, ipsilateral PCOM, ACOM, and Leptomeningeal collaterals) had excellent outcomes, indicating that as more collaterals were recruited, thus providing an effective compensatory mechanism. This finding is partially consistent with our grading system as more than 2 collaterals are important for maintaining adequate cerebral perfusion. However, in Sundaram's study, the blood flow through the ACOM alone was not essential for maintaining good cerebral perfusion.

Cheng et al. (15) studied cerebral hemodynamics using CT perfusion and collaterals using conventional angiography, and found that collateral patterns observed in TCO patients determined the extent of haemodynamic impairment. They found that patients with leptomeningeal and ophthalmic artery collaterals showed higher regional cerebral blood velocity (rCBV) and regional time to peak (rTTP) than were found in patients with collaterals through the ACOM and PCOM. Another study used selective arterial spin labeling MRI in patients with TCO and found that the presence of ipsilateral ophthalmic artery collaterals to the TCO may protect against haemodynamic impairment (16).

The reasons for the discrepancies between our results and those presented in other studies can be explained as follows. First we utilized TCCD as the only vascular imaging modality to ascertain collateral flow and pathways, while other studies used different modalities such as MR angiography (MRA) and digital subtraction angiography (DSA), for example, leptomeningeal collaterals can be detected only by DSA. Second, we involved patients with other causes of TCO in addition to atherosclerosis.

Our study has some limitations. It was a small case series, so the statistical power to detect the associations was limited. Furthermore, we didn't perform a comparative study for TCCS data with catheter-based cerebral angiography for ideal validation of our findings. Even though it is a single center study with small number of patients, we think it is good enough for a novel principle.

In summary, our study shows that it is important for collaterals to flow through the ACOM to maintain CVR in patients with TCO. Furthermore, the proposed TCCD-based collateral grading system had good reliability indices when validated against some SPECT findings, and it may therefore has good prognostic value. Although the number of patients included in this study was small, these data suggest that this Novel TCCD-based collaterals grading system could be used in CAO patients, as a non-invasive, affordable, sensitive, and convenient tool for guiding the management of these high-risk patients.

## Data Availability Statement

All datasets generated for this study are included in the article/supplementary material.

## Ethics Statement

The studies involving human participants were reviewed and approved by Ethical committee at faculty of medicine, Cairo University. The patients/participants provided their written informed consent to participate in this study. Written informed consent was obtained from the individual(s) for the publication of any potentially identifiable images or data included in this article.

## Author Contributions

All authors listed have made a substantial, direct and intellectual contribution to the work, and approved it for publication.

## Conflict of Interest

The authors declare that the research was conducted in the absence of any commercial or financial relationships that could be construed as a potential conflict of interest.
